# Statins did not reduce the frequency of exacerbations in individuals with COPD and cardiovascular comorbidities in the COSYCONET cohort

**DOI:** 10.1186/s12931-024-02822-1

**Published:** 2024-05-15

**Authors:** N. Frantzi, X. P. Nguyen, C. Herr, P. Alter, S. Söhler, D. Soriano, H. Watz, B. Waschki, F. Trinkmann, M. Eichenlaub, F. C. Trudzinski, J. D. Michels-Zetsche, A. Omlor, F. Seiler, I. Moneke, F. Biertz, G. Rohde, D. Stolz, T. Welte, H. U. Kauczor, K. Kahnert, R. A. Jörres, C. F. Vogelmeier, R. Bals, S. Fähndrich, on behalf of the German COSYCONET Cohort, Stefan Andreas, Jürgen Behr, Thomas Bahmer, Burkhard Bewig, Ralf Ewert, Beate Stubbe, HJoachim Ficker, Christian Grohé, Matthias Held, Markus Henke, Felix Herth, Anne-Marie Kirsten, Rembert Koczulla, Juliane Kronsbein, Cornelia Kropf-Sanchen, Christian Herzmann, Michael Pfeifer, JWinfried Randerath, Werner Seeger, Michael Studnicka, Christian Taube, Hartmut Timmermann, Bernd Schmeck, Hubert Wirtz

**Affiliations:** 1 Department of Pneumology, Faculty of Medicine, Medical Center, University of Freiburg, Freiburg, Germany; 2 Department of Internal Medicine V - Pulmonology, Allergology, Critical Care Care Medicine, Saarland University Hospital, Homburg, Germany; 3 Department of Medicine, Pulmonary, Critical Care and Sleep Medicine, German Center for Lung Research (DZL), Philipps University of Marburg (UMR), Marburg, Germany; 4 Airway Research Center North (ARCN), Pulmonary Research Institute at LungenClinic Grosshansdorf, Grosshansdorf, DZ Germany; 5 LungenClinic Grosshansdorf, Airway Research Center North (ARCN), Member of the German Center for Lung Research (DZL), Grosshansdorf, Germany; 6 Hospital Itzehoe, Pneumology, Itzehoe, Germany; 7 Department of Cardiology, University Heart & Vascular Center Hamburg, University Medical Center Hamburg-Eppendorf, Hamburg, Germany; 8 Department of Pneumology and Critical Care, Member of the German Center for Lung Research (DZL), Translational Lung Research Center Heidelberg (TLRC-H), Thoraxklinik Heidelberg gGmbH, Heidelberg, Germany; 9 Department of Cardiology and Angiology, Medical Center, University of Freiburg, Freiburg, Germany; 10 Department of Thoracic Surgery, Medical Center, Faculty of Medicine, University of Freiburg, Freiburg, Germany; 11 Hannover Medical School, CAPNETZ STIFTUNG, Hannover, Germany; 12 Department of Respiratory Medicine, Goethe University Frankfurt, University Hospital, Medical Clinic I, Frankfurt/Main, Germany; 13 Department of Respiratory Medicine, (BREATH), Member of the German Center for Lung Research (DZL), Research in Endstage and Obstructive Lung Disease Hannover, Hannover, Germany; 14 Diagnostic and Interventional Radiology, Member of the German Center of Lung Research, University Hospital Heidelberg, Heidelberg, Germany; 15 Department of Internal Medicine V, LMU University Hospital, LMU Munich, Comprehensive Pneumology Center, Member of the German Center for Lung Research (DZL), Ludwig-Maximilians-University Munich (LMU), Munich, Germany; 16 Institute and Outpatient Clinic for Occupational, Social and Environmental Medicine, Comprehensive Pneumology Center, Member of the German Center for Lung Research (DZL), LMU University Hospital, Ludwig-Maximilians-University Munich (LMU), Munich, Germany; 17 Helmholtz Centre for Infection Research (HZI), Helmholtz Institute for Pharmaceutical Research Saarland (HIPS), Saarland University Campus, Saarbrücken, Germany

**Keywords:** Statins, COPD, Exacerbations, Cardiovascular comorbidities

## Abstract

**Background:**

The evidence regarding effects of statins on exacerbation risk in COPD remains controversial. Previous studies often excluded patients with cardiovascular comorbidities despite their high prevalence in COPD and role for exacerbations. Based on the cardioprotective properties of statins, we hypothesised that statins may reduce the risk of exacerbations especially in patients with cardiovascular comorbidities.

**Methods:**

One thousand eight hundred eighty seven patients of the German COPD cohort COSYCONET (COPD and Systemic Consequences Comorbidities Network) of GOLD grades 1–4 (37.8% female, mean age 64.78 ± 8.3) were examined at baseline and over a period of 4.5 years for the occurrence of at least one exacerbation or severe exacerbation per year in cross-sectional and longitudinal analyses adjusted for age, gender, BMI, GOLD grade and pack-years. Due to their collinearity, various cardiovascular diseases were tested in separate analyses, whereby the potential effect of statins in the presence of a specific comorbidity was tested as interaction between statins and comorbidity. We also identified patients who never took statins, always took statins, or initiated statin intake during the follow-up.

**Results:**

One thousand three hundred six patients never took statins, 31.6% were statin user, and 12.9% initiated statins during the follow-up. Most cardiovascular diseases were significantly (*p* < 0.05)may associated with an increased risk of COPD exacerbations, but in none of them the intake of statins was a significant attenuating factor, neither overall nor in modulating the increased risk linked to the specific comorbidities. The results of the cross-sectional and longitudinal analyses were consistent with each other, also those regarding at least 1 exacerbation or at least 1 severe exacerbation per year.

**Conclusion:**

These findings complement the existing literature and may suggest that even in patients with COPD, cardiovascular comorbidities and a statin therapy that targets these comorbidities, the effects of statins on exacerbation risk are either negligible or more subtle than a reduction in exacerbation frequency.

**Trial registration:**

Trial registration ClinicalTrials.gov, Identifier: NCT01245933.

Other Study ID (BMBF grant): 01GI0881, registered 18 November 2010, study start 2010–11, primary completion 2013–12, study completion 2023–09.

https://clinicaltrials.gov/study/NCT01245933?cond=COPD&term=COSYCONET&rank=3

**Supplementary Information:**

The online version contains supplementary material available at 10.1186/s12931-024-02822-1.

## Introduction

Chronic obstructive pulmonary disease (COPD) is a heterogeneous disease with chronic airway inflammation and multi-organ involvement caused by the interplay of inhaled pollutants, genetic predisposition and socioeconomic factors [1]. Exacerbations are known to be associated with disease progression and mortality [2], thus a major treatment goal is the reduction of the frequency of exacerbations, including the possibility of drug repurposing. In the past, statins (HMG-CoA reductase inhibitors), commonly used in patients with increased cardiovascular risk, have been considered regarding this effect [3]. The reason for this is that, in addition to its specific lipid-lowering effects, this class of drugs also has pleiotropic effects, including anti-inflammatory effects, improving endothelial function, increasing the stability of atherosclerotic plaques and reducing oxidative stress [4]. Even in a population without marked hyperlipidemia (total cholesterol level < 200 mg/dL), statin intake resulted in the reduction of cardiovascular disease events [5]. Several cardiovascular conditions, such as heart failure, ischemic heart disease and atrial fibrillation, are associated with an increased risk of exacerbations and mortality in COPD [6–9], while statins are known to have positive effects on cardiovascular disease [5]. Thus, the hypothesis of an effect of statins on COPD exacerbations is well founded.

Consequently, several prospective placebo-controlled studies and cohort studies have examined the potential effect of statins on the exacerbation rate in individuals with COPD, the results, however, were equivocal [10–13]. In patients with high risk for exacerbations, for example, a prospective randomised placebo-controlled trial found no effect of 40 mg simvastatin daily on exacerbation rate or time to exacerbation [11]; however, patients taking or requiring statins due to their cardiovascular risk profile were excluded. In contrast, another study reported that simvastatin significantly prolonged the time to first COPD exacerbation and reduced the exacerbation rate [13]. Based on such findings, it was stated [14] that at present there was no evidence of a positive effect of statins on exacerbations rate and mortality but that further studies were needed.

Due to the considerations mentioned above, it seems a reasonable hypothesis that the effect of statins on exacerbation risk in COPD might only occur in the presence of cardiovascular comorbidities or cardiovascular risk factors that are targeted by this medication, and that this dependence might explain the heterogeneous data. To address this, the comprehensive set of data from patients with stable COPD of the COSYCONET (COPD and Systemic Consequences–Comorbidities Network) cohort was used.

## Methods

### Study population

The longitudinal, observational cohort COSYCONET addresses the association between chronic lung disease and comorbidities [15]. Between 2010 and 2013, 2741 individuals with stable COPD were recruited. Major inclusion criteria were age ≥ 40 years and a physician-based diagnosis of COPD; further details can be found elsewhere [15]. Assessments took part 6, 18, 36, and 54 months (Visits 2–5) after the baseline visit (Visit 1). Approval by the local ethical committees of each study center was achieved, and all patients gave their written informed consent. The study was performed according to the declaration of Helsinki, and the identifier on Clinical-Trials.gov is NCT01245933.

### Functional and clinical assessments

Details of clinical and functional assessments in COSYCONET have been described previously [15]. The measured variables included age, body mass index (BMI), smoking status, packyears, forced expiratory volume in 1 s (FEV_1_) and forced vital capacity (FVC) determined according to ATS/ERS recommendations [16], diffusing capacity for carbon monoxide (TLCO), 6-min walk distance (6-MWD) [17], the St George’s Respiratory Questionnaire (SGRQ) [18], the number of exacerbations and of severe exacerbations in the previous year; from these variables the BODE index was computed [19]. Predicted values for lung function were taken from GLI [20, 21]. In addition, the ankle-brachial (ABI) was assessed [15] and categorised according to values ≤ 0.90. Routine laboratory parameters included C-reactive protein (CRP), HbA1c [15], total cholesterol, triglycerides, and HDL- and LDL-cholesterol.

Patient-reported, physician-diagnosed comorbidities comprised diabetes, history of stroke, cardiovascular disorders including heart failure, coronary artery disease and history of myocardial infarction. All patients were categorised into GOLD groups A/B/E based on their symptoms assessed via the mMRC [22] and their exacerbation history following the criteria proposed by GOLD [2]. The documentation of the exacerbation history occurred at each visit based on the definition of acute worsening of a lung disease, such as increased shortness of breath, increased or purulent sputum. The definition of exacerbations was according to GOLD: Acute respiratory deterioration over several days and the need for specific measures (mild exacerbation: handled by the patient itself, moderate exacerbation: visit to the primary care physician, severe exacerbation: resulted in a hospital admission) [23]. Patients were asked to bring all of their medication to each study visit, which was then categorised according to ATC codes as described previously [24].

### Definition of groups and outcome

**Fig. 1:**
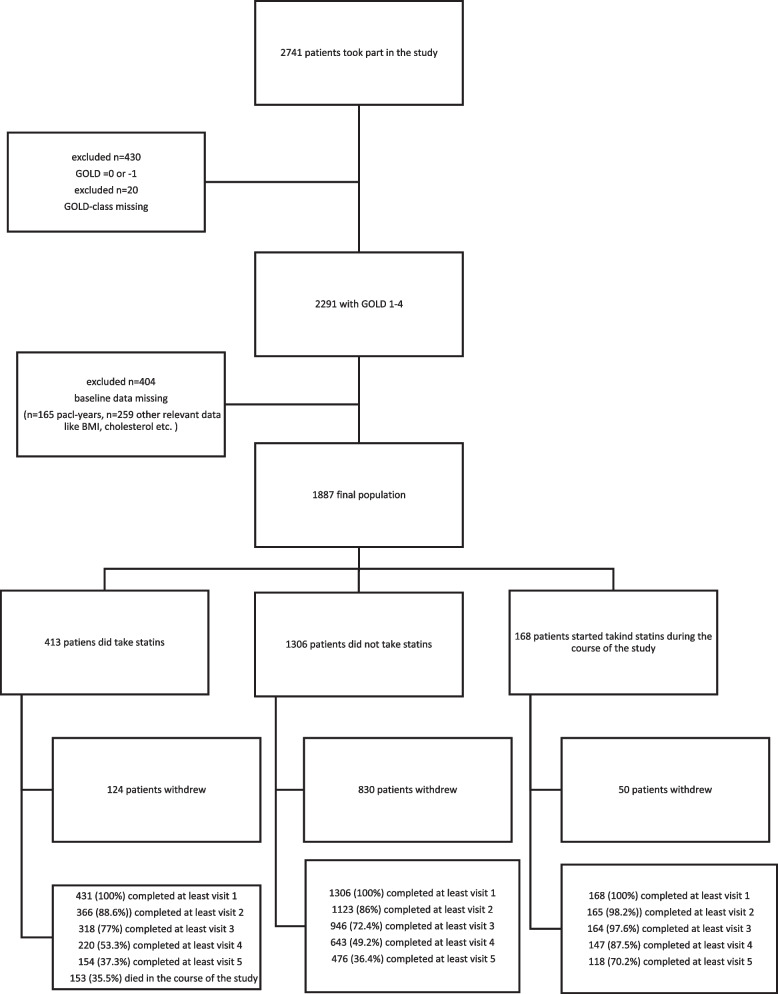
Flow chart showing the selection process of patients for analysis

We included only patients with a ratio FEV_1_/FVC < 0.7 who could be categorised into GOLD grades 1–4 according to FEV_1_ [2]. The follow-up included visits 1, 2, 3, 4 and 5, covering a period of 4.5 years (Fig. 1). For analysis, we defined the three groups of patients who were either taking statins already at baseline (statin users, SU) or statin-naïve (SN) or initiated the intake of statins during follow-up (incident-statins, IS). The endpoint of the study was the occurrence of at least one exacerbation or at least one severe exacerbation in the last year, based on the clinical history documented at each visit.

### Statistical analysis

We present categorical data as absolute counts and percentages, and continuous data as mean ± standard deviation (SD). Three types of comparison were performed. First, baseline data were compared between groups SN and SU, as well as SN and IS, using unpaired t-tests for metric variables and chi-square statistics for categorical variables. Second, we utilised logistic regression models to assess the impact of comorbidities and statins on the occurrence of at least one exacerbation per year in the baseline data, while adjusting for age, sex, BMI, GOLD grade and pack-years. Third, we employed mixed models (Generalised Estimating Equations GEE, binomial distribution, logit link) for the longitudinal analysis of statin prescription and its effect on exacerbations over visits V1 to V5 equivalent to 4.5 years of follow-up, again adjusting for age, sex, BMI, GOLD grade and pack-years; the outcomes were either the occurrence of at least one exacerbation or of at least one severe exacerbation per year. All analyses were performed separately including different cardiovascular comorbidities as predictor, due to their significant correlation with each other. Thus, besides age, sex, BMI, GOLD grade and pack-years the predictors, the logistic and the longitudinal analyses included the presence of the specific comorbidity, that of statins and an interaction term between these two factors. All patients with all their visits were kept in the longitudinal analyses but additionally a sensitivity analysis was performed including only those patients that remained in the study until visit 5. A two-sided *p*-value < 0.05 was considered as statistically significant. For data analysis, the software package SPSS (Version: 29.0.0.0., IBM, Armonk, NJ, USA) was used.

## Results

### Baseline characteristics

Figure 1 illustrates the inclusion and follow-up criteria of the patients. 450 patients were excluded. Patients with FEV1/FVC ≥ 0.7 (*n* = 430) dropped out. 20 patients had no available information on their GOLD class. 234 patients were not considered due to missing information on their pack-years. 127 patients were excluded due to missing information on LDL cholesterol. The remaining exclusions were attributed to missing information in various parameters: BMI: 2 patients, Exacerbations: 3 patients, FEV1: 16 patients, TLCO: 160 patients, CRP: 41 patients, HbA1c: 71 patients. Out of the 413 patients in the SU group, 154 (37.3%) completed visit 5. Similarly, among the 1306 patients in the SN group, 476 (36.4%) completed visit 5. Table 1 shows the baseline characteristics of the three groups.

**Table 1:** Baseline characteristics

	**Statin naive (SN) *n* = 1306**	**Statin users (SU) *n* = 413**	**Incident-statins (IS) *n* = 168**	***p*-value SN vs SU**	***p*-value SN vs IS**	***P*-value SU vs IS**
Age (y)	63.8 ± 8.6	67.5 ± 7.3^a^	66.2 ± 7.0^b^	< 0.001	< 0.001	0.044
Height (10)	170.7 ± 9.2	172.3 ± 8.2^a^	171.5 ± 9.1	< 0.001	0.271	0.352
Sex (m/f)	755 (57.8%)	308 (74.6%)^a^	111 (66.1%)^b^	< 0.001	0.046	< 0.001
BMI (kg/m^2^)	26.42 ± 5.43	27.45 ± 4.95^a^	26.96 ± 4.6	< 0.001	0.163	0.253
Pack-years	47.21 ± 34.2	55.5 ± 39.1^a^	54.5 ± 40.3^b^	< 0.001	0.025	0.791
FEV_1_ (% predicted)	52.2 ± 18.3	53.7 ± 18.9	59.6 ± 18.3^b^	0.147	< 0.001	< 0.001
FVC (% predicted)	78.7 ± 18.7	77.9 ± 19.3	84.6 ± 18.9^b^	0.445	< 0.001	< 0.001
FEV_1_/FVC	0.66 ± 0.14	0.68 ± 0.14^a^	0.70 ± 0.14^b^	< 0.001	< 0.001	< 0.001
TLCO (% predicted)	51.7 ± 20.1	53.6 ± 20.6	57.3 ± 21.8^b^	0.110	0.002	0.112
BODE index > 4 (*n* = 1297)	149 (11.4%)	53 (12.8%)	17 (10.1%)	0.342	0.241	0.843
6MWD (m)	419.0 ± 107.7	401.1 ± 108.9^a^	446.4 ± 87.3^b^	0.001	< 0.001	< 0.001
Cardiovascular disease ≥ 2	155 (11.9%)	196 (47.5%)^a^	37 (22.0%)^b^	< 0.001	< 0.001	< 0.001
Diabetes mellitus	119 (9.11%)	105 (25.42%)^a^	19 (11.31)	< 0.001	0.397	< 0.001
Asthma	213 (16.3%)	59 (14.3%)	25 (14.9%)	0.354	0.738	0.595
History of stroke	30 (3.0%)	36 (8.7%)^a^	5 (3.0%)	< 0.001	1.0	< 0.001
Heart failure	45 (3.45%)	42 (10.17%)^a^	10 (5.95%)	< 0,001	0,126	< 0.001
Cardiac arrhythmias (SN = 651, SU = 206, IS = 87)	96 (14.75%)	41 (19.90%)	16 (18.39)	0.082	0.425	1.183
Peripheral artery disease	122 (9.34%)	79 (19.13%)^a^	20 (11.9%)	< 0.001	0.270	< 0.001
Coronary artery disease	107 (8.19%)	160 (38.74%)^a^	29 (17.26%)^b^	< 0.001	< 0.001	< 0.001
Hypertension	643 (48.55%)	299 (72.40%)^a^	101 (60.11%)^b^	< 0.001	0.005	< 0.001
Moderate exac. prev. year ≥ 1	480 (36.8%)	151 (36.6%)	51 (30.4%)	0.953	0.068	0.262
Severe exac. prev. year ≥ 1	260 (19.9%)	94 (22.8%)	24 (14.3%)^b^	0.301	0.020	0.660
SGRG total	42.9 ± 19.9	43.9 ± 19.9	40.6 ± 18.8	0.159	0.147	0.029
Total Cholesterol (mmol/L)	5.67 ± 1.14	4.87 ± 1.21^a^	4.78 ± 1.16^b^	< 0.001	< 0.001	0.390
HDL-cholesterol (mmol/L)	1.78 ± 0.60	1.61 ± 0.60^a^	1.67 ± 0.52^b^	< 0.001	0.012	0.222
LDL-cholesterol (mmol/L)	3.39 ± 0.97	2.73 ± 1.01^a^	2.6. ± 1^b^	< 0.001	< 0.001	0.321
Triglycerides (mmol/L)	1.5 ± 0.96	1.7 ± 1.11^a^	1.53 ± 0.82	< 0.001	0.689	0.04
HbA1c (%)	5.84 ± 0.62	6.14 ± 0.83^a^	5.86 ± 0.55	< 0.001	0.702	< 0.001
CRP (mg/L)	10.9 ± 27.2	9.2 ± 19.67	8.4 ± 21.9	0.167	0.186	0.694
GOLD grade 1–4	159/572/471/104 12.2/43.8/36.1/9.0%	52/187/144/30 12.6/45.3/ 34.9/7.3%	29/94/40/5^b^ 17.3/56.0/ 23.8/3.0%	0.505	0.001	0.001
GOLD group A/B/E	171/653/480 13.1/50.1/ 36.8%	55/207/151 13.3/50.1/ 36.6%	28/89/51 16.7/53.0/30.4%	0.904	0.067	0.495

*BMI* body-mass index, *FEV*_*1*_ forced expiratory volume in 1 s, *FVC* forced vital capacity, *TLCO* transfer factor for carbon monoxide, *6MWD* 6-min walking distance, BODE index composed of body-mass index, airflow obstruction, dyspnea and 6MWD; *SGRQ* St George’s Respiratory Questionnaire, *HDL* high-density lipoprotein, *LDL* low-density lipoprotein, *HbA1c* glycated hemoglobin, *CRP* C-reactive protein, *GOLD* Global Initiative for Chronic Obstructive Lung Disease

Patient characteristics of the three statin groups of the longitudinal cohort at baseline (visit 1). Depending on the type of variable and the data distribution, mean values ± standard deviations or numbers and percentages (in parentheses) are given. P values refer to comparisons between the two groups indicated using either the unpaired t-test (when mean values are given) or chi-square statistics (when categorical data are given) at an overall significance level of 0.05. The asterisks give the additional information whether the respective comparison was also significant after applying a Bonferroni adjustment factor of 2, as only two pairwise comparisons were made

^a^never-statins vs always-statins

^b^never-statins vs incident-statins

#### Statin users (SU) versus statin-naïve (SN)

There were significant differences between the two groups. More men (74.6% vs. 57.8%) and more patients with diabetes (25.4 vs. 9.1%) and stroke (8.7 vs. 3.0%) took statins from the beginning until visit 5, compared to the individuals who were statin-naïve. Furthermore, subjects in the SU group were older (67.5 ± 7.3 vs. 63.8 ± 8.6 years), had higher BMI (27.5 ± 5.0 vs. 26.4 ± 5.4 kg/m^2^), and exhibited significantly lower LDL cholesterol levels (2.73 ± 1.01 vs. 3.39 ± 0.97), lower HDL cholesterol levels (1.61 ± 0.6 vs. 1.78 ± 0.6), lower total cholesterol levels (4.87 ± 1.21 vs. 5.67 ± 1.14), higher triglyceride levels (1.70 ± 1.11 vs. 1.50 ± 0.96), and higher HbA1c levels (6.14 ± 0.83 vs. 5.84 ± 0.62). The two groups did not show significant differences with regard to FEV_1_% predicted, FVC % predicted, SQRQ, the distribution of GOLD grades, BODE index, and CRP levels.

#### Incident-statins (IS) versus statin-naïve (SN)

Compared to the SN group, statins were newly prescribed during the follow-up (IS) in older subjects (66.2 ± 7.0 vs. 63.8 ± 8.6) and individuals with more packyears of smoking (54.5 ± 40.3 vs. 47.2 ± 34.2). These subjects also had a better lung function in terms of FEV_1_% predicted (59.6 ± 18.3 vs. 52.2 ± 18.3) and higher FVC % predicted (84.6 ± 18.9 vs. 78.7 ± 18.7). At baseline, total cholesterol was lower (4.78 ± 1.16 vs. 5.67 ± 1.14), LDL cholesterol was lower (2.60 ± 1.0 vs. 3.39 ± 0.97), and HDL cholesterol was lower (1.67 ± 0.52 vs. 1.78 ± 0.60). BMI, prevalence of diabetes, history of stroke, SQRQ, BODE index, HbA1c and CRP did not significantly differ between the two groups.

### Association between statin intake, comorbidities and exacerbation frequency

Overall, 7777 acute COPD exacerbations were registered during the 4.5-year follow-up. Table 2 displays the distribution of exacerbations across visits. In the unpaired t-test there were no significant differences in exacerbation rates per person year between the SU and the SN groups, with values of 1.2 ± 1.87 vs. 1.22 ± 1.87, respectively (*p* = 0.87).

**Table 2:** Number,mean of exacerbations and number of patients (in parentheses) at each study visit

	**V1**	**V2**	**V3**	**V4**	**V5**
Statin users (SU)	538 (*n* = 413) 1.30	332 (*n* = 366) 0.91	287 (*n* = 317) 0.91	358 (*n* = 220) 1.63	144 (*n* = 152) 0.95
Statin naïve (SN)	1742 (*n* = 1306) 1.33	1114 (*n* = 1122) 0.99	995 (*n* = 946) 1.05	755 (*n* = 643) 1.17	529 (*n* = 476) 1.11
Incident-statins (IS)	159 (*n* = 168) 0.95	167 (*n* = 165) 1.01	291 (*n* = 164) 1.77	199 (*n* = 147) 1.35	167 (*n* = 118) 1.42

Number of acute exacerbations at each study visit V1-V5

*n* = number of patients at each visit. The percentages refer to the proportion of exacerbations based on the number of patients

#### Cardiovascular diseases and exacerbation frequency at baseline

**Fig. 2:**
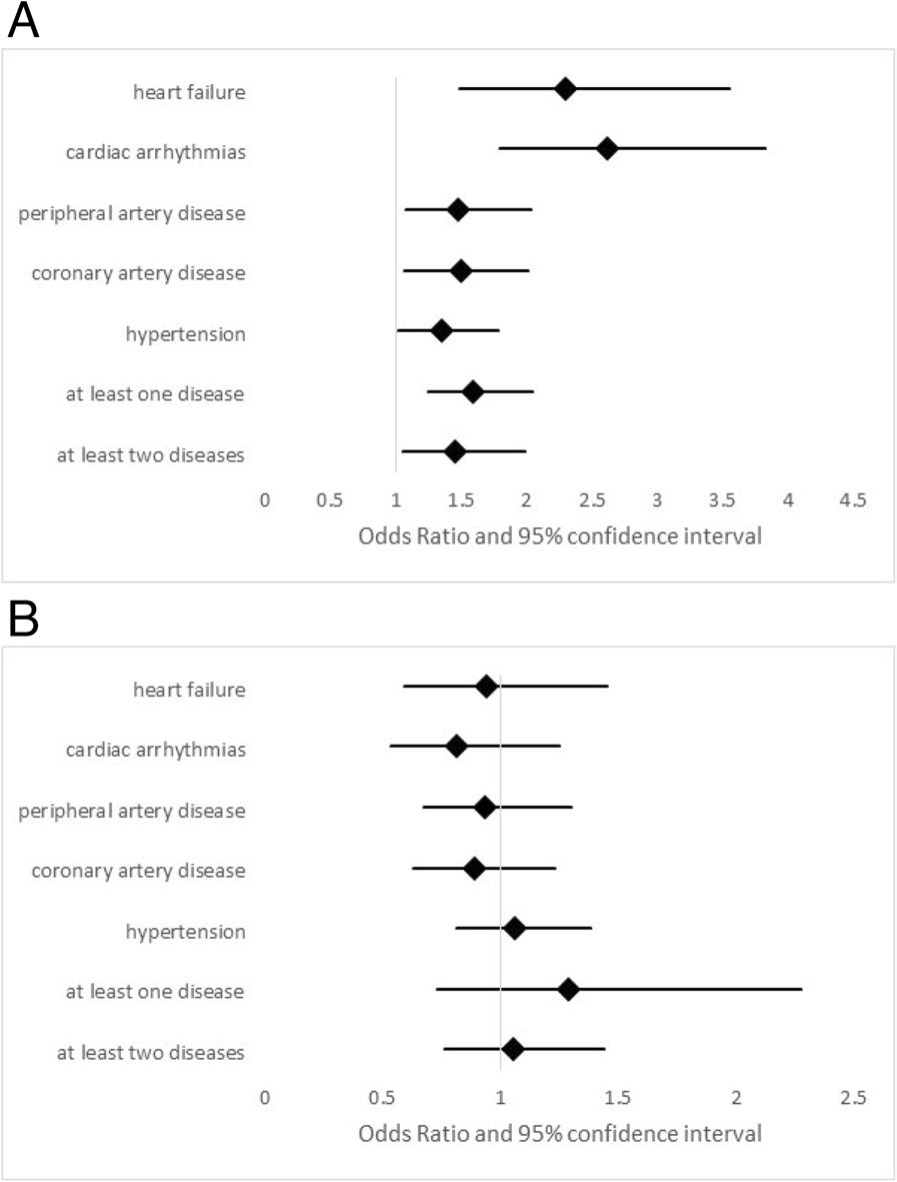
**A** Forest plot displaying the adjusted odds ratios and their 95% confidence intervals for the association between each cardiovascular comorbidity (and a summary value for at least one disease and a summary value for the combination of two comorbidities) and the occurrence of at least one exacerbation per year at baseline. The numerical data can be found in Table 3 and were obtained by logistic regression analysis adjusted for sex, age, BMI, GOLD grade and pack-years.** B** Forest plot displaying the adjusted odds ratios and their 95% confidence intervals for the association between the medication with statins and the occurrence of at least one exacerbation per year at baseline for each of the cardiovascular comorbidities (and a summary value for at least one disease and a summary value for the combination of two comorbidities) shown in Fig. 2A. The numerical data can be found in Table 3 and were obtained by logistic regression analysis adjusted for sex, age, BMI, GOLD grade and pack-years

We investigated whether cardiovascular comorbidities were associated with an elevated risk of exacerbations, using baseline data and logistic regression models. All five cardiovascular entities that had been included were significantly associated with the exacerbation risk, with Odds Ratios as shown in Table 3 and illustrated in Fig. 2A. The presence of statins did not show any significant associations with exacerbations (Table 3, Fig. 2B), and the same was true for the interaction term between statins and comorbidity (Table 3), suggesting the absence of differential effects in patients with specific cardiovascular comorbidities.

**Table 3:** Association between the occurrence of at least 1 exacerbation per year and several cardiovascular comorbidities as well as statins in the baseline data

	**Comorbidity**	**Statins**	**Interaction comorbidity-statin**
	**OR (95% CI) *p*-value**	**OR (95% CI) *p*-value**	**OR (95% CI) *p*-value**
**Heart failure**	2.29 (1.48; 3.55) < 0.001	0.94 (0.59; 1.45) 0.73	1.32 (0.57; 3.06) 0.51
**Cardiac arrhythmias**	2.62 (1.79; 3.83) < 0.001	0.81(0.53; 1.25) 0.35	1.41(0.59; 3.33) 0.44
**Peripheral artery disease**	1.47 (1.07; 2.03) 0.02	0.93 (0.67; 1.30) 0.68	0.85 (0.44; 1.61) 0.61
**Coronary artery disease**	1.49 (1.06; 2.01) 0.02	0.89 (0.63; 1.23) 0.47	1.21(0.66; 2.23) 0.54
**Hypertension**	1.35 (1.02; 1.78) 0.037	1.06 (0.81; 1.38) 0.68	1.11 (0.58; 2.10) 0.76
**At least 1 disease**	1.59 (1.24; 2.04) < 0.001	1.29 (0.73; 2.28) 0.39	0.75 (0.40; 1.41) 0.37
**At least 2 diseases**	1.45 (1.05; 1.99) 0.024	1.05 (0.76; 1.44) 0.78	0.92 (0.55; 1.55) 0.76

**Fig. 3:**
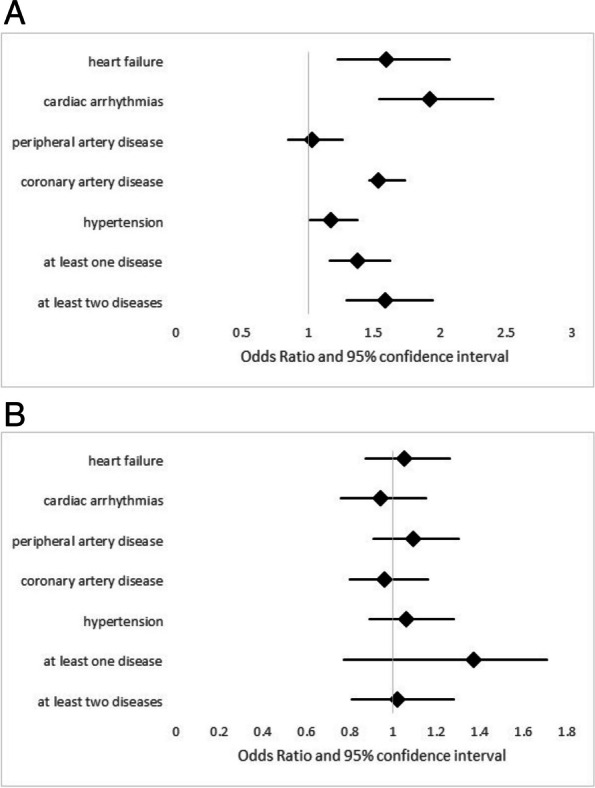
**A** Forest plot displaying the adjusted odds ratios and their 95% confidence intervals for the association between each cardiovascular comorbidity (and a summary value for at least one disease and a summary value for the combination of two comorbidities) and the occurrence of at least one exacerbation per year between the investigated cardiovascular diseases and exacerbation occurrence during the 4.5 years of follow up. The numerical data can be found in Table 4 and were obtained by a GEE analysis adjusted for sex, age, BMI, GOLD grade and pack-years.** B** Forest plot displaying the adjusted odds ratios and their 95% confidence intervals for the association between the medication with statins and the occurrence of at least one exacerbation per year at baseline for each of the cardiovascular comorbidities (and a summary value for at least one disease and a summary value for the combination of two comorbidities) shown in Fig. 3A during the 4.5 years of follow up. The numerical data can be found in Table 4 and were obtained by a GEE analysis adjusted for sex, age, BMI, GOLD grade and pack-years

Results of logistic regression analyses on the occurrence of at least one exacerbation, using the whole set of baseline data (visit V1). As predictors each analysis comprised one of the comorbidities, the presence of statins and an interaction term between these two factors, as well as the covariates age, sex, BMI, GOLD grade and pack-years. The table shows the odds ratios (OR) and their 95% confidence intervals (95% CI) regarding comorbidities (left column), statins (middle column) and the interaction (right column) for each of the analyses. The odds ratios are visualized in Figs. 2 and 3

#### Cardiovascular diseases and exacerbation frequency in the follow-up

In the longitudinal analysis over 4.5 years, the occurrence of at least one exacerbation per year, heart failure, cardiac arrhythmias, CAD and hypertension showed significant associations with the occurrence of exacerbations, while PAD did not (Table 4, Fig. 3A). Statins did not have significant effects in any of the cardiovascular conditions (Table 3, Fig. 3B), and the same applied to the interaction terms (Table 4).

**Table 4:** Association between the occurrence of at least 1 exacerbation per year and several cardiovascular comorbidities as well as statins in the follow-up data

	**Comorbidity**	**Statins**	**Interaction comorbidity-statins**
	**OR (95% CI) *p*-value**	**OR (95% CI) *p*-value**	**OR (95% CI) *p*-value**
**Heart failure**	1.59 (1.22; 2.07) 0.001	1.05 (0.87; 1.26) 0.62	1.79 (0.47; 1.34) 0.38
**Cardiac arrhythmias**	1.92 (1.54; 2.40) 0.001	0.94 (0.76; 1.15) 0.52	0.80 (0.49; 1.29) 0.36
**Peripheral artery disease**	1.03 (0.85; 1.26) 0.76	1.09 (0.91; 1.30) 0.37	0.76 (0.50; 1.14) 0.18
**Coronary artery disease**	1.53 (1.46; 1.73) 0.001	0.96 (0.80; 1.16) 0.68	1.27 (0.83; 1.93) 0.27
**Hypertension**	1.17(1.01; 1.37) 0.04	1.06 (0.89;1.28) 0.50	0.84 (0.58; 1.22) 0.35
**At least 1 disease**	1.37 (1.16; 1.62) < 0.001	1.37 (0.77; 1.71) 0.50	0.88 (0.56; 1.36) 0.56
**At least 2 diseases**	1.58 (1.29; 1.94) < 0.001	1.02 (0.81; 1.28) 0.88	0.88 (0.63; 1.24) 0.48

Results of (logistic) GEE analyses on the occurrence of at least one exacerbation, using the whole set of data during the course of 4.5 years (visits V1-V5). As predictors each analysis comprised one of the comorbidities, the presence of statins and an interaction term between these two factors, as well as the covariates age, sex, BMI, GOLD grade and pack-years. The table shows the odds ratios (OR) and their 95% confidence intervals (95% CI) regarding comorbidities (left column), statins (middle column) and the interaction (right column) for each of the analyses. The odds ratios are visualized in Figs. 3A and B

**Fig. 4:**
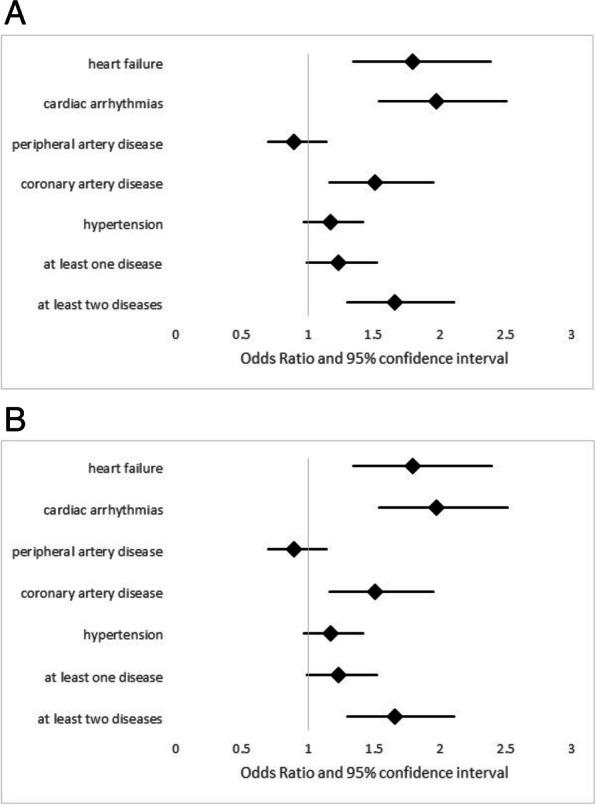
**A** Forest plot displaying the adjusted odds ratios and their 95% confidence intervals for the association between each cardiovascular comorbidity and the occurrence of at least one severe exacerbation per year between the investigated cardiovascular diseases and exacerbation occurrence during the 4.5 years of follow up (and a summary value for at least one disease and a summary value for the combination of two comorbidities). The numerical data can be found in Table 5 and were obtained by a GEE analysis adjusted for sex, age, BMI, GOLD grade and pack-years.** B** Forest plot displaying the adjusted odds ratios and their 95% confidence intervals for the association between the medication with statins and the occurrence of at least one severe exacerbation per year at baseline for each of the cardiovascular comorbidities shown in Fig. 4A during the 4.5 years of follow up (and a summary value for at least one disease and a summary value for the combination of two comorbidities). The numerical data can be found in Table 5 and were obtained by an GEE analysis adjusted for sex, age, BMI, GOLD grade and pack-years

When repeating the analysis with the outcome of at least one severe exacerbation per year, qualitatively similar results were obtained as for exacerbations in general (Table 5, Figs. 4A and B), with the exception that hypertension was no more significantly associated with an increased exacerbation risk. It also became apparent that only the presence of at least 2 cardiovascular comorbidities was significantly associated with increased risk of severe exacerbation.

**Table 5:** Association between the occurrence of at least 1 severe exacerbation per year and several cardiovascular comorbidities as well as statins in the follow-up data

	**Comorbidity**	**Statins**	**Interaction comorbidity-statins**
	**OR (95% CI) *p*-value**	**OR (95% CI) *p*-value**	**OR (95% CI) *p*-value**
**Heart failure**	1.79 (1.34; 2.39) 0.001	1.05 (0.85; 1.31) 0.64	0.78 (0.44; 1.38) 0.39
**Cardiac arrhythmias**	1.97 (1.54; 2.51) < 0.001	0.99 (0.78; 1.27) 0.96	0.88 (0.51; 1.51) 0.64
**Peripheral artery disease**	0.89 (0.7; 1.14) 0.34	1.14 (0.92; 1.41) 0.25	1.01 (0.61; 1.68) 0.96
**Coronary artery disease**	1.51 (1.16; 1.95) 0.002	0.99 (0.78; 1.26) 0.94	0.93 (0.55; 1.55) 0.77
**Hypertension**	1.17 (0.97; 1.42) 0.10	1.09 (0.88; 1.36) 0.41	0.92 (0.58; 1.47) 0.73
**At least 1 disease**	1.23 (0.99; 1.52) 0.06	1.17 (0.70; 1.96) 0.55	0.91 (0.52; 1.59) 0.74
**At least 2 diseases**	1.66 (1.30; 2.11) < 0.001	1.12 (0.83; 1.52) 0.45	0.76 (0.50; 1.17) 0.21

Results of (logistic) GEE analyses on the occurrence of at least one severe exacerbation, using the whole set of data during the course of 4.5 years (visits V1-V5). As predictors each analysis comprised one of the comorbidities, the presence of statins and an interaction term between these two factors, as well as the covariates age, sex, BMI, GOLD grade and pack-years. The table shows the odds ratios (OR) and their 95% confidence intervals (95% CI) regarding comorbidities (left column), statins (middle column) and the interaction (right column) for each of the analyses. The odds ratios are visualized in Figs. 4A and B

In order to reveal whether the result critically depended on patients’ loss to follow-up, we performed a sensitivity analysis. For this purpose, the longitudinal analyses were repeated while including only those patients who participated until visit 5. The results of this sensitivity analysis for at least one exacerbation are given in the Supplemental Table S1 and those for at least one severe exacerbation in the Supplemental Table S2. It can be seen that only heart failure and cardiac arrhythmias were significantly related to the occurrence of at least one exacerbation or one severe exacerbation and that in case of severe exacerbations the presence of at least 2 comorbidities was required for a significant association. Statins still did not show significant associations.

## Discussion

In this study, we examined potential effects of statins on exacerbation risk in patients with COPD, focusing on the potential role of cardiovascular comorbidities for such effects. Given the high prevalence of cardiovascular comorbidities in COPD and the fact that a number of previous studies did exclude patients with cardiovascular disease [11, 13], this seems of both scientific and clinical interest. The present study confirmed the results of previous cross-sectional analyses in the COSYCONET cohort that cardiovascular comorbidities were associated with an increased risk of exacerbations [25, 26]. In line with this, it also demonstrated in a longitudinal analysis over a 4.5-year period that the presence of heart failure, cardiac arrhythmias, coronary artery disease and hypertension was associated with exacerbation risk. Patients with COPD often show cardiovascular comorbidities that can be the cause of an acute deterioration independent of the lung disease [25, 26]. This association could be based on several factors, a fact that was covered by the rather general (until 2022) definition of exacerbations as “acute worsening of respiratory symptoms that results in additional therapy” which did not address specific causes [27]. Statins have their place in the prevention of cardiovascular diseases [28–30]. Thus, it appears reasonable to expect effects on COPD exacerbation risk in subjects with at least one cardiovascular comorbidity. Contrary to our hypothesis, however, there was no reduction in the risk of exacerbations including severe exacerbations. Even in patients with at least two cardiovascular comorbidities, statins did not have a significant effect in the longitudinal follow-up compared to patients not taking statins. This also applied to patients who got their first prescribed statins during the observation period of 4.5 years. We used cross-sectional and longitudinal statistical standard approaches including main factors and interaction terms, but neither the main factors of statin intake nor their interactions with cardiovascular comorbidities indicated an effect.

Our data are in line with those of the multi-centre placebo-controlled study by Criner et al., in which simvastatin at a daily dose of 40 mg had no effect on the rate of exacerbations and the time to the next exacerbation [11]. This study was performed in 885 patients with COPD treated from 12 to 36 months, who were at high risk for exacerbations but without cardiovascular diseases, diabetes and without requiring statins. These findings and our own findings are, however, not in line with those of the recently published prospective, randomised, double-blinded, placebo-controlled study by Schenk et al. investigating the impact of simvastatin on exacerbation rate and time to next exacerbation [13]. It was found that 40 mg simvastatin daily prolonged the time to first exacerbation and reduced the exacerbation rate. As the authors excluded patients with cardiovascular diseases, this seems remarkable, if it is argued that effects were to be expected primarily in these patients. There are, however, differences in sample size and follow-up period that might be relevant. While in COSYCONET subjects were observed over a follow-up period of 4.5 years, the scope of the prospective randomised study was limited to 1 year. During this time, 66 subjects with placebo and 72 subjects on simvastatin completed the study. On the other hand, a clear strength was that Schenk et al. used diaries that enabled a precise recording and reporting of exacerbations.

Although the available data therefore remain conflicting, our study adds to the current knowledge as it particularly addressed the hypothesis that the effects of statins would be apparent in those patients with COPD who had cardiovascular diseases and thus a risk factor for exacerbations that was targeted by the statins. The observed lack of effect suggests that the effects of statins on acute cardiovascular events that are related to exacerbations were secondary, despite the well-known long-term protective effect of statins on the progression of cardiovascular disease. Nevertheless, in the future, a randomized controlled study would be beneficial which specifically examines the effect of statins on cardiovascular events and exacerbations in patients with COPD and cardiovascular diseases.

### Limitations and strengths of the study

COSYCONET is not a placebo-controlled, randomised study, and its observational character naturally limits the scope of conclusions that can be drawn. Moreover, although the definition of exacerbations followed established criteria and practice, it certainly would have benefitted from a more precise recording, for example via an electronic diary. On the other hand, at least the occurrence of severe exacerbations can be assumed to have been recorded reliably. We also cannot provide data on the association between statin intake and the precise time to the next exacerbation (time to event). Moreover, we analysed statins as a drug class without distinguishing between different drugs of this class, whereas the randomised studies examined specific drugs, in particular simvastatin, and it cannot be excluded that effects differ between different statins. Furthermore, we did not have information about the duration of intake until inclusion in the cohort study, and the different dosages and types of preparations could not be taken into account. It should also be noted that we cannot exclude a healthy survivor bias due to loss to follow-up, although the comparison of all patients with those remaining in the study until the end of follow-up yielded consistent results. In the longitudinal analyses reported here, we included visit 2 data despite their overlap with visit 1 data, as sensitivity analyses omitting visit 2 data did not yield different results. Moreover, in the study population visits were performed throughout the year, thus potential inhomogeneity over time due to the fact that exacerbations were reported for the previous 12 months and study visits were mostly separated by 18 months, are unlikely. The strengths of our study are that we examined a large number of subjects over a long period of time and covered a whole range of cardiovascular comorbidities that allowed for a detailed analysis of potential effects of statins in specific cardiovascular conditions.

## Conclusion

The present study addressed the hypothesis that in patients with COPD the presence of cardiovascular comorbidities might be a relevant factor determining the effect of statins on exacerbation risk. Using a large data set, we found in cross-sectional and longitudinal analysis that most cardiovascular comorbidities were associated with increased exacerbation risk but that statins were neither linked to an overall reduction of this risk nor to specific effects in patients with different cardiovascular comorbidities. These data complement the existing literature and may suggest that even in patients with a therapy that specifically targets such comorbidities, potential effects of statins are either negligible or more subtle than a reduction of exacerbation frequency. Additional research is required to expand upon this hypothesis in future studies.

### Supplementary Information

Additional file 1:** Supplemental Table S1:** Results of (logistic) GEE analyses on the occurrence of at least one exacerbation using data over the course of 4.5 years (visits V1-V5). Only patients who maintained in the study until visit V5 were included. As predictors each analysis comprised one of the comorbidities, the presence of statins and an interaction term between these two factors, as well as the covariates age, sex, BMI, GOLD grade and pack-years. The table shows the odds ratios (OR) and their 95% confidence intervals (95% CI) regarding comorbidities (left column), statins (middle column) and the interaction (right column) for each of the analyses.** Supplemental Table S2: **Results of (logistic) GEE analyses on the occurrence of at least one severe exacerbation using data over the course of 4.5 years (visits V1-V5). Only patients who maintained in the study until visit V5 were included. As predictors each analysis comprised one of the comorbidities, the presence of statins and an interaction term between these two factors, as well as the covariates age, sex, BMI, GOLD grade and pack-years. The table shows the odds ratios (OR) and their 95% confidence intervals (95% CI) regarding comorbidities (left column), statins (middle column) and the interaction (right column) for each of the analyses.

## Data Availability

No datasets were generated or analysed during the current study.
