# Supplementary material for: Statins did not reduce the frequency of exacerbations in individuals with COPD and cardiovascular comorbidities in the COSYCONET cohort
Source: Respir Res. 2024 May 15;25:207. doi: 10.1186/s12931-024-02822-1 (PMC11097413; doi:10.1186/s12931-024-02822-1)
Supplement: Supplementary file 1 — Additional file 1: Supplemental Table S1: Results of (logistic) GEE analyses on the occurrence of at least one exacerbation using data over the course of 4.5 years (visits V1-V5). Only patients who maintained in the study until visit V5 were included. As predictors each analysis comprised one of the comorbidities, the presence of statins and an interaction term between these two factors, as well as the covariates age, sex, BMI, GOLD grade and pack-years. The table shows the odds ratios (OR) and their 95% confidence intervals (95% CI) regarding comorbidities (left column), statins (middle column) and the interaction (right column) for each of the analyses. Supplemental Table S2: Results of (logistic) GEE analyses on the occurrence of at least one severe exacerbation using data over the course of 4.5 years (visits V1-V5). Only patients who maintained in the study until visit V5 were included. As predictors each analysis comprised one of the comorbidities, the presence of statins and an interaction term between these two factors, as well as the covariates age, sex, BMI, GOLD grade and pack-years. The table shows the odds ratios (OR) and their 95% confidence intervals (95% CI) regarding comorbidities (left column), statins (middle column) and the interaction (right column) for each of the analyses. [file 12931_2024_2822_MOESM1_ESM.docx]

**Supplemental Table S1**

|  | **Comorbidity** | **Statins** | **Interaction comorbidity-statins** |
| --- | --- | --- | --- |
|  | **OR (95% CI) p-value** | **OR (95% CI) p-value** | **OR (95% CI) p-value** |
| **Heart failure** | 1.94 (1.27; 2.95) 0.002 | 0.85 (0.63; 1.15) 0.29 | 0.65 (0.32; 1.31) 0.23 |
| **Cardiac arrhythmias** | 1.71 (1.18; 2.48) 0.005 | 0.80 (0.58; 1.11) 0.18 | 1.13 (0.57; 2.24) 0.73 |
| **Peripheral artery disease** | 0.97 (0.66; 1.42) 0.86 | 0.90 (0.66; 1.22) 0.49 | 0.85 (0.46; 1.56) 0.60 |
| **Coronary artery disease** | 1.35 (0.84; 2.16) 0.22 | 0.64 (0.45; 0.91) 0.012 | 1.63 (0.82; 3.23) 0.16 |
| **Hypertension** | 1.96 (0.92; 1.52) 0.19 | 1.05 (0.65;1.70) 0.85 | 0.71 (0.40; 1.27) 0.25 |
| **At least 1 disease** | 1.27 (0.998; 1.63) 0.052 | 0.71 (0.40; 1.28) 0.26 | 1.15 (0.61; 2.18) 0.66 |
| **At least 2 diseases** | 1.36 (0.98; 1.88) 0.07 | 0.77 (0.54; 1.09) 0.14 | 1.03 (0.60; 1.76) 0.91 |

**Supplemental Table S2:**

|  | **Comorbidity** | **Statins** | **Interaction comorbidity-statins** |
| --- | --- | --- | --- |
|  | **OR (95% CI) p-value** | **OR (95% CI) p-value** | **OR (95% CI) p-value** |
| **Heart failure** | 2.18 (1.27; 3.74) 0.005 | 0.94 (0.63; 1.40) 0.76 | 0.49 (0.21; 1.14) 0.10 |
| **Cardiac arrhythmias** | 2.37 (1.57; 3.59) <0.001 | 0.83 (0.53; 1.29) 0.40 | 0.97 (0.41; 2.28) 0.95 |
| **Peripheral artery disease** | 0.63 (0.38; 1.04) 0.71 | 0.91 (0.62; 1.33) 0.62 | 1.37 (0.62; 3.02) 0.44 |
| **Coronary artery disease** | 1.61 (0.93; 2.80) 0.09 | 0.85 (0.53; 1.36) 0.50 | 0.87 (0.37; 2.05) 0.75 |
| **Hypertension** | 1.25 (0.91; 1.73) 0.17 | 1.05 (0.55; 1.97) 0.90 | 0.79 (0.36; 1.72) 0.55 |
| **At least 1 disease** | 1.35 (0.97; 1.88) 0.07 | 1.17 (0.58; 2.35) 0.66 | 0.70; 0.32; 1.52) 0.36 |
| **At least 2 diseases** | 1.59 (1.01 ;2.34) 0.02 | 0.99 (0.61; 1.62) 0.97 | 0.65 (0.31; 1.37) 0.26 |
